# Extracellular vesicles from infected cells: potential for direct pathogenesis

**DOI:** 10.3389/fmicb.2015.01132

**Published:** 2015-10-20

**Authors:** Angela Schwab, Shabana S. Meyering, Ben Lepene, Sergey Iordanskiy, Monique L. van Hoek, Ramin M. Hakami, Fatah Kashanchi

**Affiliations:** ^1^Laboratory of Molecular Virology, School of Systems Biology, George Mason University, Manassas, VA, USA; ^2^School of Nursing and Health Studies, Georgetown University, Washington, DC, USA; ^3^Ceres Nanosciences, Inc., Manassas, VA, USA

**Keywords:** exosome, virus, extracellular vesicle, bacteria, protozoa, pathogen, parasite

## Abstract

Infections that result in natural or manmade spread of lethal biological agents are a concern and require national and focused preparedness. In this manuscript, as part of an early diagnostics and pathogen treatment strategy, we have focused on extracellular vesicles (EVs) that arise following infections. Although the field of biodefense does not currently have a rich resource in EVs literature, none the less, similar pathogens belonging to the more classical emerging and non-emerging diseases have been studied in their EV/exosomal contents and function. These exosomes are formed in late endosomes and released from the cell membrane in almost every cell type *in vivo*. These vesicles contain proteins, RNA, and lipids from the cells they originate from and function in development, signal transduction, cell survival, and transfer of infectious material. The current review focuses on how different forms of infection exploit the exosomal pathway and how exosomes can be exploited artificially to treat infection and disease and potentially also be used as a source of vaccine. Virally-infected cells can secrete viral as well as cellular proteins and RNA in exosomes, allowing viruses to cause latent infection and spread of miRNA to nearby cells prior to a subsequent infection. In addition to virally-infected host cells, bacteria, protozoa, and fungi can all release small vesicles that contain pathogen-associated molecular patterns, regulating the neighboring uninfected cells. Examples of exosomes from both virally and bacterially infected cells point toward a re-programming network of pathways in the recipient cells. Finally, many of these exosomes contain cytokines and miRNAs that in turn can effect gene expression in the recipient cells through the classical toll-like receptor and NFκB pathway. Therefore, although exosomes do not replicate as an independent entity, they however facilitate movement of infectious material through tissues and may be the cause of many pathologies seen in infected hosts.

## Extracellular Microvesicles

Small membrane bound vesicles secreted by cells can be detected using electron microscopy (Delabranch et al., 2012). These extracellular vesicles (EVs) are released by all eukaryotic cells studied to date as evident from both *in vitro* as well as *in vivo* studies. They are implicated to have roles in cell-cell communication through protein and nucleic acid transfer, but also in carrying biomarkers of disease (Fleming et al., 2014). The EV proteome, lipidome and mRNA/miRNAome represent a snapshot in time of the cell status at the moment of release. These EVs can carry bioreactive macromolecules such as nucleic acids, proteins, and lipids, and therefore may contribute to pathogenesis.

### Types of Microvesicles

Various small vesicles, discovered in the 1960s, were originally considered to be small platelets less than 1 μm wide (Delabranch et al., 2012). There are several types of these extracellular microvesicles, many of which are discussed in this review. Microparticles are formed by ectocytosis, budding of the cell membrane (Delabranch et al., 2012; Lagana et al., 2013), and are generally 50–2000 nm (Akers et al., 2013). Retrovirus-like particles (RLPs) are 90–100 nm and non-infectious, and they are released from cells after a viral infection. Apoptotic bodies are the largest group of microvesicles, 500–4000 nm, but smaller vesicles are also formed when a cell undergoes apoptosis, 50–500 nm. The different types of microvesicles are differentiated by their cellular origin and not so much by size since their sizes tend to overlap.

The word “exosome” was coined by Dr. Rose Johnstone because they seemed to undergo “reverse endocytosis” (Johnstone et al., 1987; Akers et al., 2013). Exosomes are defined as membrane-bound vesicles formed within late endosomes and secreted from the cell (Delabranch et al., 2012; Akers et al., 2013; Lagana et al., 2013). Exosomes are usually 30–100 nm in length and contain both functional and as yet undefined proteins and RNAs. Bacterial cells also secrete vesicles, typically in the range of 10–300 nm in diameter (MacDonald and Kuehn, 2012), and are often induced under conditions of membrane stress. Vesicles that take in outside material through macropinocytosis and enter the endosomal pathway are different and called intraluminal vesicles (ILVs). Most of these types of vesicles are formed by clathrin at the cell membrane (Nour and Modis, 2014). Table 1 shows a list of the types of EVs discussed, their sizes, and their protein markers.

**TABLE 1:** **Types and sizes of membrane vesicles**.

**Type of membrane vesicle**	**Size**	**Marker**	**Reference**
Microparticles	50–2000 nm	VCAMP3, ARF6	Akers et al. (2013), Ghossoub et al. (2014), Kalasin and Santore (2015)
Retrovirus-like particles (RLPs)	75–100 nm	Gag	Roberts et al. (2012), Akers et al. (2013)
Apoptotic bodies	500–4000 nm	Thrombospondin, C3b, Annexin V	Flügel-Koch et al. (2002), Akers et al. (2013), Cheung et al. (2015), Roy et al. (2015)
Bacterial extracellular vesicles	10–300 nm	PAMPs	Bhatnagar et al. (2007), MacDonald and Kuehn (2012), Brown et al. (2014), Fleming et al. (2014)
Exosomes	30–100 nm	Transferrin, CD9, CD63, CD81, TSG101, Alix	Schorey and Bhatnagar (2008), Subra et al. (2010), Delabranch et al. (2012), Akers et al. (2013), Ghossoub et al. (2014), Raimondo et al. (2015)

### Intraluminal Membrane Vesicles

Intraluminal vesicles are part of the endosomal pathway. They take in components of the cellular membrane and recycle biological material in a process called “back-fusion” (Nour and Modis, 2014). Several viruses can use back-fusion as a route to infecting cells, as the ILVs can take the virus particles straight to the nucleus (Lagana et al., 2013; Nour and Modis, 2014). The first virus to be discovered using back fusion as a delivery method into the cytosol is the vesicular stomatitis virus (Nour and Modis, 2014). Back fusion is most beneficial to DNA viruses that require entry into the nucleus in order to replicate, though there is evidence that some members of *Flavivirus* genus also use this pathway to infect cells. *Bacillus anthracis* also utilizes back-fusion as a means of delivering toxins into the recipient cells (Nour and Modis, 2014).

### Bacterial Membrane Vesicles

Similar to eukaryotic cells, bacteria also make and release membrane vesicles. Gram-negative bacteria commonly are found to produce vesicles, called outer-membrane vesicles that derive from “blebbing” of the outer membrane, and forming vesicles that can contain proteins, membrane components and occasionally nucleic acids. Of the gram-negative bacteria that produce these vesicles, many are pathogenic (Kadurugamuwa and Beveridge, 1999; Pierson et al., 2011; Nieves et al., 2014) and can have toxic effects on host cells (Pierson et al., 2011), or can deliver antigens and thus act as a potential vaccine (Pierson et al., 2011; Nieves et al., 2014). Recently, gram-positive bacteria have also been observed to produce vesicles (Brown et al., 2014), although the mechanism may be different compared to gram-negative organisms (Haurat et al., 2014). These bacterial vesicles may have roles in both intra-species and inter-species communications (Berleman and Auer, 2013), as well as potential inter-kingdom interaction with the host (Kulp and Kuehn, 2010; Pierson et al., 2011). Finally, these vesicles provide a new approach for development of non-live vaccines, and for instance have been successfully used in a New Zealand study with children infected with *Neisseria meningitidis* (Wong et al., 2009).

### Purification Methods

**FIGURE 1:**
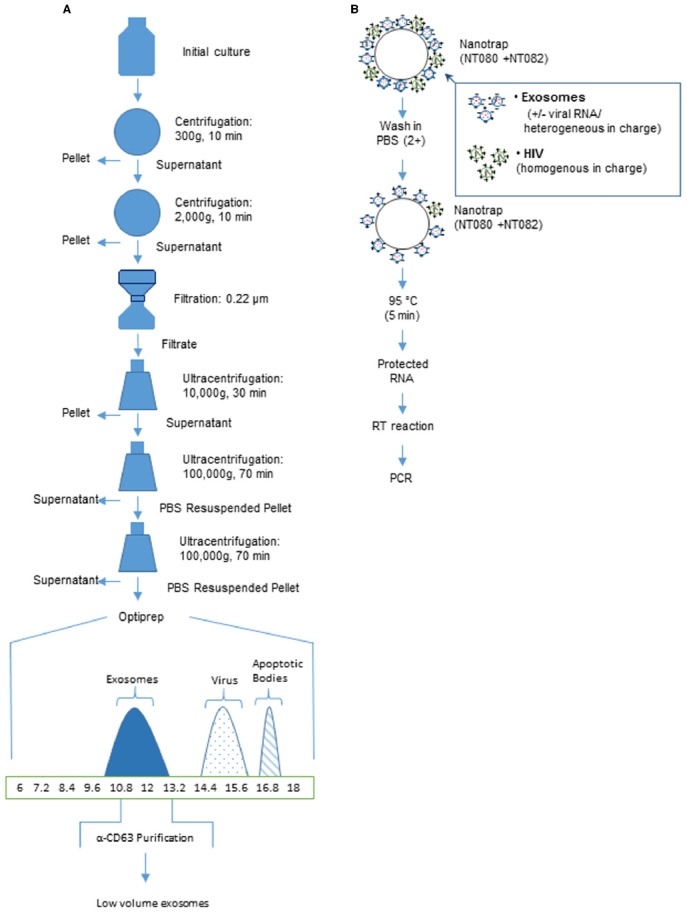
**Purification of exosomes using centrifugation or affinity beads. (A)** An overview of a generalized procedure utilizing multistep centrifugation for high volume of culture medium. A culture of infected cell supernatants (∼100 ml grown in exosome free media) is centrifuged at multiple speeds and for varying times. The samples are next filtered to remove large debris including dead cells. After the final ultracentrifugation, the sample represents partially purified exosomes (∼80%). Exosomes can be further purified using sucrose density or OptiPrep gradient (>95%). The x-axis represents % iodixanol in OptiPrep gradients. These fractions can then be further purified with magnetic beads coated with antibodies to exosomal proteins such as C63 [usually present in higher amounts from RNA virus infected cells such as HIV, HTLV-1, RVFV and Ebola]. **(B)** An overview of low volume purification with Nanotrap particles to separate exosomes from the virus particles. Nano-captured exosomes can be used for PCR analysis (modified from Sampey et al., 2014).

Whether they will be used as therapeutic agents or for research purposes, it is necessary to purify exosomes using precise and reproducible techniques (Thery et al., 2006). Exosomes are present in low concentrations in extracellular fluids. Purification begins with large volumes of cell-free exosome-containing fluids to which increasing centrifugal forces are applied (Akers et al., 2013). The ensuing pellet is then further purified over a sucrose gradient and then immunoprecipitated using antibodies to known exosomal markers, as viruses may co-purify with the exosomal prep obtained from the gradient (Delabranch et al., 2012; Akers et al., 2013; Figure 1). For *in vitro* analysis, many novel approaches can be used prior to the centrifugation step. For instance the analysis of secretory proteins from the cell line HepG2 and human liver slices, utilized metabolic labeling of proteins which was carried out for brief time periods, followed by collection and filtration of cell supernatants and subsequent protein precipitation and analysis using sensitive biochemical methods (Zwickl et al., 2005).

Since the commonly used methods are time-consuming, many colleagues in the field are actively working toward more time-saving isolation methods. Kanwar et al. (2014) have developed a three-step immunoaffinity method using a microvesicle-chip coated with antibodies against CD63. Clayton et al. (2001) have developed a similar technique using magnetic bead isolation. The use of antibodies in both methods renders the isolated exosomes non-functional. Therefore, such methods of exosomal preparation may only be used in diagnostic testing and not so much for functional studies of exosomes. Another novel technique for detecting microvesicles is through μNMR (Akers et al., 2013). It is more precise than western blotting and ELISA as it can distinguish between microvesicles from healthy samples vs. cancer samples. It would be interesting to utilize μNMR in diseases such as viral and bacterial infections since they also secrete vesicles that are smaller and have different membraneous composition.

To discriminate exosomes and other small extracellular particles, including virions, Jaworski et al. (2014a,b) proposed to apply innovative Nanotrap technique. Nanotrap particles are a novel technology that can address most critical analytical challenges for pathogen identification and measurement. They are homogenous hydrogel particles of about 800 nm in size that have a shell made of polymers of *N*-isopropylacrylamide (NIPAm) and co-monomers such as acrylic acid (AAc) and allylamine (AA) with cross links of *N*,*N*′-methylenebisacrylamide (BIS; Luchini et al., 2008, 2009). Charge-based affinity baits are also incorporated into the shell of the particles (Luchini et al., 2009). The recently performed screening of Nanotrap particles indicated that the particles containing vinyl sulfonic acid (VSA) in shell were highly efficient at capture of Rift Valley fever virus (RVFV; Shafagati et al., 2013) and HIV-1 virions (Jaworski et al., 2014b), whereas the Nanotrap particles comprised of a shell architecture without VSA were able to selectively capture exosomes from both cell culture media and serum (Jaworski et al., 2014b). The exact mechanism of this binding is unknown, although the selective capturing of the viral particles and exosomes may be associated with their different surface charge or various glycosylation of surface glycoproteins.

#### Biomarkers of Microvesicles

The microvesicle biomarkers that have been cataloged include proteins, mRNA, miRNA, and lipids. Various databases provide a rich resource for the study of exosome components. Recently, there have been several new databases developed to collect the proteomic data that has been generated through proteomics analysis of exosomes. These databases include EVPedia (Kim et al., 2013), Vesiclepedia (Kalra et al., 2012), and Exocarta (Kalra et al., 2012) among others. EVPedia has cataloged almost 50,000 proteins found in exosomes, more than 164,000 mRNAs, and 12,000 miRNAs in various types of membrane vesicles. Vesiclepedia has collected data from more than 350 different studies. When examining these databases, there appears to be few common proteins identified in these exosomes including the exosome marker proteins Alix, CD63, CD81, CD9 (Schorey and Bhatnagar, 2008), transferrin, and TSG101 (Akers et al., 2013; Table 1). Although most of the published literature relies on these proteins, there may be varying levels of certain markers including TSG101, Alix, and CD63 following an infection. Finally, each of the different types of microvesicles have certain proteins and molecules that can be used as specific markers, including thrombospondin, C3b, and Annexin V (for detection of apoptotic bodies), as well as VCAMP3 and ARF6 (for detection of microparticles; Akers et al., 2013). Overall, more than 52,000 proteins have been observed in microvesicles, ∼165,000 mRNAs, ∼13,000 miRNAs, and ∼350 different lipid species across the multiple databases.

### Nucleic Acid Component of Exosomes

Recently, miRNAs have been found outside of cells (Chen et al., 2013; Lagana et al., 2013), circulating in serum and saliva, and can be associated with microvesicles and exosomes (Gallo et al., 2012). Some cell types are able to transcribe miRNAs for the purpose of secretion through exosomes, which can then be used to show phenotypic changes in recipient cells (Lagana et al., 2013). The gold standard technique for analyzing circulating miRNAs within exosomes and EVs from serum or plasma is qRT-PCR (Moldovan et al., 2013). Exosome-centric studies of mRNA and miRNA have led to the “Trojan Horse” hypothesis, as a potential new method of genetic exchange between infected cells and uninfected neighbor cells (Gould et al., 2003; Valadi et al., 2007). miRandola and miRiam are two programs available to predict and evaluate miRNA in the circulation and from specific pathogens; RepTar, ViTa, and vHoT are a few databases containing miRNA information and predictions (Lagana et al., 2013). These are used to determine what kind of an effect, if any, that a viral genome will have on target cells (Lagana et al., 2013).

A virally-infected cell can secrete cellular and viral miRNA via exosomes (Pegtel et al., 2010). Viral infection may affect the composition of cellular miRNAs that are secreted by the infected cell, and thus indirectly alter cell–cell communication (Wurdinger et al., 2012). For instance, DNA and RNA viruses mediate carcinogenesis via their encoded miRNAs (Verweij et al., 2013). HIV-1 has at least two known miRNAs, one from TAR element and the other called hiv1-mir-H1, which causes host cells to undergo apoptosis (Lagana et al., 2013). Most of the viruses that code miRNAs are DNA viruses, mainly herpesviruses (Lagana et al., 2013).

### Exosome Lipids

Microvesicles are secreted as a result of signal transduction. An influx of calcium activates two phospholipid transporters, scramblase and floppase, which send phosphatidylserine to the outer leaflet of the membrane where the microvesicle will bud (Subra et al., 2010; Delabranch et al., 2012; Nour and Modis, 2014). The lipid composition of the vesicles differ from that of the parent cell (Delabranch et al., 2012). Some viruses, such as *Flavivirus*, can cause calcium to flow into the host cell (Nour and Modis, 2014). Most microvesicles have receptors and adhesion molecules to signal target cells (Delabranch et al., 2012), and can transfer these signals either via surface receptors or by endocytosis of the vesicle by the target cell (Nour and Modis, 2014). One example is tissue factor being brought to neutrophils and platelets. Exosomes can interact with CD91 and Tim4 when attaching to recipient cells (Subra et al., 2010).

### Pathogens Use Exosomes for Their Immediate Spread

Pathogens use exosomes to spread infection and to avoid the host immune system (Delabranch et al., 2012; Lagana et al., 2013). Viral infections can enter the latent phase using exosomes to safely spread their miRNAs, undetectable to the immune system (Lagana et al., 2013). The virus can hide in a few cells, slowly causing neighboring cells to become more vulnerable to infection through the miRNAs, until a signal triggers the virus to replicate again and thus cause a rapid infection. It is not yet fully understood how the viruses use microvesicles to avoid detection by the immune system. It is thought that the immune system cannot detect miRNAs of non-host origin, allowing the virus to spread rapidly when using exosomes to transport viral miRNAs (Lagana et al., 2013). For instance, the Chagas parasite transmits exosomes into the host’s bloodstream to deactivate C3 convertase of the complement pathway (Delabranch et al., 2012); and hepatitis C infected cells effectively utilize exosomes to infect other cells, where exosomes are not recognized by the host innate or acquired immune responses (Nour and Modis, 2014).

### Exosomes and the Immune System

The immune system uses toll-like receptors (TLRs) to recognize pathogenic foreign nucleic acid (Chen et al., 2013). When triggered, TLRs can cause a cell to release cytokines to attract phagocytes to the location. TLR3 (Bai et al., 2014; Jiménez-Sousa et al., 2015), TLR7 (Cohen et al., 2015), TLR8 (Cohen et al., 2015), and TLR9 all are capable of binding to viral dsRNA and ssRNA as well as viral and bacterial DNA (Chen et al., 2013; Ishii et al., 2014). TLR8, more specifically, has been shown to bind to ssRNA including HIV-1 genomic RNA (Cohen et al., 2015). MiRNAs have recently been found to bind to TLRs as well. TLR8 on macrophages can bind to miR-21 and miR-29a that are released from tumors, and thus cause proinflammatory cytokine release from these cells. TLR7 can bind to let-7b on neurons, leading to neurodegeneration *in vitro* and *in vivo* (Winkler et al., 2014). These TLRs bind to GU-rich regions of the miRNA, but the exact mechanism for the interaction is currently unknown (Chen et al., 2013). TLRs of dendritic cells (DCs), T cells, and macrophages activate these cells when they come into contact with exosomes containing viral miRNA (Chen et al., 2013; Nour and Modis, 2014). This may contribute to novel vaccine methods to target exosomes where antibodies may be ineffective (Lagana et al., 2013; Nour and Modis, 2014).

Exosomes can also carry MHC class I and MHC class II molecules and cause immunosuppression (Yang et al., 2012b). Exosomes, especially from DC, have been shown to reduce inflammation in mice due to IL-10, B7, and MHC class II. Exosomes from plasma in mice can contain other protein markers such as CD71, FasL (also aids in suppressing inflammation), and CD86 (Yang and Robbins, 2012). Exosomes from tumor cells can contain transforming growth factor-β (TGF-β) on the lipid surface to suppress T cells (Tran et al., 2015). Many cytokines in macrophage-derived exosomes, such as CCL3, CCL4, CCL5, TNFα, G-CSF, CSCL2, and IL-1RA, cause immune stimulation in target cells. Pathogen-associated molecular patterns (PAMPs) are also packaged into the exosomes from bacterially-infected cells to cause a faster immune response (McDonald et al., 2014). Exosomes from B cells can contain LAMP-1, CD20, BCR, various tetraspanins, as well as heat shock proteins (Gehrmann et al., 2014). Overall, immunoregulatory molecules incorporated into the exosomes may cause both immuno-suppressive and immuno-activation effects depending on their composition and cellular source of the exosomes.

#### CD4^+^ Cells

T helper cells that express the CD4 glycoprotein on their surface are also known as CD4^+^ T cells. Activation of CD4^+^ T cells occurs when they are presented with peptide antigens complexed to MHC class II molecules. It was thought that such presentation occurred when peptides are expressed on the surface of antigen-presenting cells (APCs) such as DCs. However, evidence is accumulating to show that even exosomes secreted by DCs induce stimulation of CD4^+^ T cells *in vivo* and *in vitro* (Thery et al., 2002; Matsumoto et al., 2004; Hao et al., 2007). Activated CD4^+^ T cells release exosomes that contain T cell receptors (TCRs) on their surface that stimulate exosome release from activated B cells (Choudhuri et al., 2014). In turn, B-cell-released exosomes can directly stimulate primed (but not naïve) CD4^+^ T cells, to create a feedback loop in the process of T cell/B cell collaboration (Muntasell et al., 2007).

Furthermore, secretion of exosomes from CD4^+^ T cells as well as other immune cells also occurs constitutively. Secretion of cytokines and their receptors, as well as other protective factors occurs via exosomes. Documented cytokines and their receptors include tumor necrosis factor-α (TNFα), TNFαR, TGF-β, interleukin-1β (IL-1β), IL-18, IL-32, and death receptors ligands such as FASL or TNF-related apoptosis-inducing ligand (TRAIL; Gutierrez-Vazquez et al., 2013). For example, chicken biliary exosomes possess the capacity to influence the immune responses of lymphocytes. Specifically, they promote the proliferation of CD4^+^ and CD8^+^ T cells and monocytes from liver and inhibit the oncogenic retrovirus, avian leukosis virus subgroup J, from replicating *in vitro* (Wang et al., 2014). The converse also occurs where CD4^+^ T cell proliferation and cytokine secretion can be suppressed by transforming miRNAs from exosomes derived from T-reg cells (Okoye et al., 2014).

Exosomes secreted by CD4^+^ T cells protect uninfected cells from viral infection and also protect them from reactivation of viral elements contained in exosomes. The key factor within exosomes mediating viral deactivation may be the nucleic acid editing APOBEC3 (A3) family of cellular cytidine deaminases (Khatua et al., 2009). This family of cytidine deaminase enzymes is known to provide protection against reactivation of Alu and L1 retrotransposons. Khatua et al. (2010) have demonstrated that exosomal A3G and A3F proteins and A3G mRNA secreted by CD4^+^H9 T cells and mature monocyte-derived DCs were functional *in vitro* to inhibit L1 and Alu retrotransposition. Collectively, exosomes encapsulate factors (proteins and RNA) that are mostly functional in protecting neighboring cells against reactivation of viral elements by forming a secondary line of defense following viral entry.

#### Exosomes as Vaccines

Exosomes have been shown to have the potential to be used as vaccines. For instance, *Streptococcus pneumonia*-infected dendritic-cell derived exosomes contain capsular polysaccharide (capsular antigen) and protect mice against *S. pneumonia* infection (Colino and Snapper, 2007). *Toxoplasma gondii* infected DC-derived exosomes were used to protect against *T. gondii* infection in mice (Beauvillain et al., 2009). DC derived exosomes from cells infected with the parasite *Eimeria* were found to convey protection in a poultry model (del Cacho et al., 2013). DC derived exosomes were also found to confer protective immunity against *Leishmania* in mice (Schnitzer et al., 2010). Exosomes from B cells infected by Epstein-Barr virus (EBV) protect uninfected B-cells from EBV infection (Vallhov et al., 2010), and exosomes from *Leishmania*-infected macrophages confer protection to naïve macrophages (Cronemberger-Andrade et al., 2014). Khatua et al. (2009) have demonstrated that exosomes containing A3G provide uninfected T cells resistance to continued HIV-1 proliferation. The prevalence of DC and macrophage-derived exosomes in these examples reveals an important and direct interaction of exosomes with the immune system. Therefore DC derived exosomes are a major source of potential vaccines (Pitt et al., 2014).

As with any vaccine, there are limitations to using exosomes. One limitation is that they are heterogeneous, containing various proteins and materials from cells, making the exosomes harder to control than liposomes. Currently, liposomes are used as drug- and antigen-delivery vessels because they are protective of the cargo, can carry hydrophobic and hydrophilic molecules, and they can cross the plasma membrane into cells easily (Lai et al., 2013). Liposomes are a useful tool, but they can prove toxic in some instances (Lai et al., 2013). Liposomes are easy to synthesize and to package the immunogens and adjuvants, whereas exosomes have proven to be much more difficult for insertion of desired components (Lai et al., 2013). It is important to note that exosomes injected into a patient must compete with natural exosomes in the body to have the desired effects. Manipulating the lipid composition and ligands/receptors on exosomes can decrease the uptake of exosomes from specific cell types. Recent studies have shown that exosomes from DCs can be blocked from entering bone marrow derived dendritic cells (BMDC) by coating the exosome’s CD11a, CD54, CD9, or CD81 with antibodies. Exosomes can also be blocked by binding of competitive ligands to the cell receptors (Marcus and Leonard, 2013).

#### Exosomes as Therapeutics

Biotherapeutics range from small peptides to siRNA molecules. In the laboratory, they are highly efficacious. In the clinic, they have shown their limitations with respect to lower half-lives, off-target effects and inability to cross the blood–brain barrier. Exosomes can help overcome these drawbacks. Since exosomes are natural agents of intercellular delivery, they can be exploited to become carriers of engineered therapeutics (Kooijmans et al., 2012). Some drugs can be inserted into purified exosomes *in vitro*, or incorporated into exosomes as they are made *in vivo*, by tagging the drugs for exosomal targeting (Lai et al., 2013). Exosomes that can target certain tissue types and can protect cargo from the immune system are more ideal than liposomes, as they also have a longer half-life (Lai et al., 2013).

Sun et al. (2010) provided the first proof-of-concept for biotechnological exploitation of exosomes. Curcumin was mixed with exosomes to enhance the bioavailability, stability, and solubility (Sun et al., 2010). Mixing with exosomes improved the anti-inflammatory activity compared with curcumin alone in an *in vivo* lipopolysaccharide-induced septic shock model (Sun et al., 2010). A subsequent study by (Zhuang et al., 2011) went a step further by utilizing exosomes to deliver anti-inflammatory drugs to the brain through a non-invasive intranasal route. These exosomes were designed to enclose either curcumin or a Stat3-inhibitor; both molecules were able to bypass the blood–brain barrier.

Exosomes can target to specific tissue types naturally and artificially. Astrocytes can release miR-29b in exosomes after the cells are given morphine and Tat from HIV-1. Cultured neurons treated with these exosomes displayed miR-29b in their cytoplasm within 4 h. This miRNA targets the gene PDGF-B and causes neuronal death. It is well known that astrocytes communicate with neurons, and this study demonstrated first that the exosomes play a key role in this communication (Hu et al., 2012). Exosomes can participate in neuron synapses to promote survival and growth of target neurons. Another study focused on analyzing the effects of exosome-incorporated siRNA, showing that injections of the vesicles into blood of laboratory animals can in turn stop gene expression in CNS (Gupta and Pulliam, 2014). The exosomes derived from bone marrow stromal cells are important for communication with multiple myeloma cells, and promote growth, migration, and drug resistance. These exosomes transfer such cytokines as IL-6, MCP-1, and IL-13 (Wang et al., 2014).

Experiments by Alvarez-Erviti et al. (2011) included a homing mechanism to their exosomes so as to specifically target a certain tissue type where the exosomes would unload their cargo. Exosomes derived from immature DCs were used for delivering exogenous siRNA *in vitro* as well as *in vivo* by, i.v., injection to target the brain. Targeting was achieved by engineering the exosomal surface protein lamp2b to also display a targeting peptide derived from the rabies virus glycoprotein (RVG). This peptide is known to bind nicotinic acetylcholine receptor present on neurons and the vascular endothelium of the blood–brain barrier. The method proved efficacious with up to 60% RNA and protein knockdown predominantly in the neurons of the midbrain, cortex and striatum, and this method was neither toxic nor immunogenic. Taken together, these examples demonstrate the potential of exosomes to be used for targeted delivery of multiple therapeutics to different tissues and cell types.

## Infections and Exosomes

**FIGURE 2:**
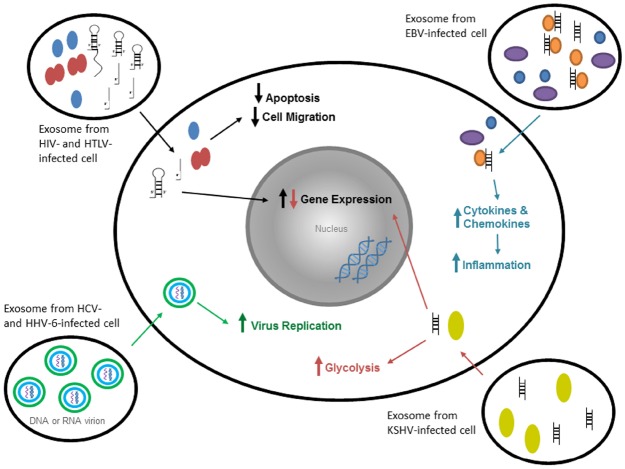
**Exosomes from virally-infected cells have various specific effects on target cells.** Exosomes from HIV-1- and HTLV-1-infected cells contain viral and cellular proteins and double- and single-stranded RNA. Within a target cell, the proteins decrease apoptosis and cell migration, whereas RNA increases gene expression in the nucleus in order to promote infection. Exosomes from HCV-infected cells contain non-enveloped virions (both mature and immature). These virions can enter a target cell using exosomes and replicate on the ER membranes. Exosomes from HHV-6-infected cells contain mature virions that spread infection faster through the use of exosomes. Exosomes from EBV-infected cells contain viral DNA and viral and cellular proteins that increase cytokine and chemokine production in target cells (through innate immune molecules), and thus increase cytokine expression and inflammation. Exosomes from KSHV-infected cells contain viral miRNAs and proteins that increase glycolysis and gene expression in target cells.

Fungi, bacteria, protozoa, and viruses all secrete some form of microvesicle (Delabranch et al., 2012). For instance, viral infections cause exosome formation and secretion from infected host cells. HIV-1 infected cells releases negative factor (Nef) protein in exosomes to alter dynamics of signal transduction in host recipient cell (Delabranch et al., 2012; Lagana et al., 2013). Hepatitis C and Hepatitis A infected cells use exosomes as a vesicle to infect new cells with viral RNA and proteins. Some non-enveloped viruses, such as Hepatitis A, use the host cell’s endosomes to envelope the virus (Nour and Modis, 2014). Figure 2 shows some effects of exosomes from virally-infected cells on uninfected target cells. Finally, non-nucleic acid infections material (such as prions) have been identified in exosomes which may contribute to pathogenesis (Fevrier et al., 2004; Leblanc et al., 2006; Saá et al., 2014). Cells infected with bacteria can release exosomes that presents PAMPs to immune cells (Bhatnagar et al., 2007). Protozoan parasites also have been shown to release exosomes with protozoan molecules into the bloodstream (Beauvillain et al., 2009). Therefore, in the following section, we will describe some of the highlights from the literature on how exosomes containing viral, bacterial, and other agents contribute to pathogenesis.

### DNA Viruses

#### Epstein-Barr Virus

The EBV is a large DNA virus associated with B-cell lymphomas and carcinomas in tongue, nasopharynx, and stomach. The virus is found in a latent stage in both lymphomas and carcinomas (Morales-Sanchez and Fuentes-Panana, 2014). The presence of oncoproteins and viral miRNAs in exosomes from EBV-infected cells gives credence to the fact that virus-infected cells use the exosomal pathway to regulate gene expression in the surrounding tissue to avert destruction by the immune system. Evidence is accumulating to show that inhibitory exosomes promote immune evasion and progression of EBV associated tumors such as Hodgkin’s disease (HD; Gandhi et al., 2007) and nasopharyngeal carcinoma (NPC; Li et al., 2007). Besides EBV, other DNA and RNA viruses also mediate carcinogenesis via their encoded miRNAs (Verweij et al., 2013).

Meckes et al. (2013) studied the specific effects of the oncogenic herpesviruses, EBV and Kaposi sarcoma-associated virus, on the proteomes of B-cell exosomes using global quantitative proteomics. They studied exosomes purified from 11 B-cell lines that were uninfected, infected with EBV or with Kaposi’s sarcoma-associated herpesvirus (KSHV), or infected with both viruses. Using mass spectrometry, 871 proteins were identified, of which ∼360 were unique to the viral exosomes. Analysis by 2D difference gel electrophoresis and spectral counting indicates that the viruses greatly impact the protein content of exosomes with common and distinct changes induced by both viruses. Of the proteins found in samples from EBV and KSHV exosomes, several have similar functions associated with cancer, predicting disorders and disease, cell survival or cell death, and signaling of EIF2 in the canonical pathway. It is likely that these alterations in exosome content modulate the tumor environment, potentially to enhance viral infection and promote tumorigenesis (Meckes et al., 2013).

EBV-transformed cells express at least 44 mature viral miRNAs that target viral and cellular genes along with deregulating the miRNA profile of the host cell. MiRNA profiling showed that 2% of the miRNAs of diffuse large B-cell lymphoma (DLBCL) and nasal NK/T-cell lymphoma (NKTL) are derived from the virus, while 20% of the total miRNA in NPC are viral miRNAs (Barth et al., 2011). The miRNAs inhibit the apoptotic response of the infected cell as a means to establish a latent infection. Other studies have corroborated the observation that besides oncoproteins, miRNA encoded by EBV may also contribute to EBV-mediated transformation of B-cells (Feederle et al., 2011; Vereide et al., 2014).

***Biogenesis and uptake of exosomes***

Epstein-Barr virus exploits the endosomal–exosomal pathway to enclose both EBV-associated and cellular macromolecules in the endosomes. MiRNAs secretion via exosomes begins with their assembly with RISC at endosomes (Pegtel et al., 2011). Thus miRNA function, mRNA stability and exosome-mediated intercellular communication converge at the level of endosomes (Verweij et al., 2011).

CD63, found in exosomes, is a tetraspanin protein that assembles in small membranous platforms and guides intracellular trafficking of associated-partners. LMP1 co-opts the physiological role of CD63 in human B cells for its own benefit. Knock-down of CD63 by short hairpin RNAs against CD63 led to sequestering of LMP1 protein in intracellular compartments; so, LMP1 may associate with CD63 prior to reaching the plasma membrane (Verweij et al., 2011).

Released exosomes act on neighboring as well as distant cells by circulating through plasma. They are internalized by recipient cells via caveolae-dependent endocytosis and eventually traffic through an endosomal pathway (Nanbo et al., 2013). The transferred molecules are functional in recipient cells. For example LMP1 released in this manner causes the upregulation of ICAM-1 in recipient cells (Nanbo et al., 2013). These results are in line with a previous publication showing that LMP1 induces the expression of cellular proteins, including ICAM-1 in EBV-infected cells (Wang et al., 1990). Internalized exosomes lead to isolated B-cell’s proliferation, induction of activation-induced cytidine deaminase, and the production of circle and germline transcripts for IgG1 (Gutzeit et al., 2014). In this manner, some or all of the exosomal factors may be at play in preventing uninfected B cells from being infected by EBV, as demonstrated for B cells isolated from umbilical cord blood (Vallhov et al., 2010). EBV studies suggest that exosomes derived from different cells types have different mechanisms for targeting recipient cells. For example, exosomes derived from EBV-transformed B cell lines target B-cells; this targeting can be blocked efficiently by anti-CD21 or anti-gp350, molecules present on B-cells and exosomes derived from EBV infected cells respectively (Vallhov et al., 2010).

***EBV proteins in exosomes***

EBV-infected B cells secrete exosomes containing: EBV-encoded miRNAs (Pegtel et al., 2010), mRNA for EBNA (Canitano et al., 2013), small non-coding RNA complexed with proteins (Ahmed et al., 2014) and various proteins of viral origin such as LMP1 (Meckes et al., 2010; Yoshizaki et al., 2013), and host origin such as galectin-9 (Gourzones et al., 2012). Host originating proteins include pro-tumorigenic molecules including HIF1α (Aga et al., 2014).

LMP1 is one of the best recognized oncogenic viral protein. It is a signaling protein that imitates a constitutively active TNF receptor. In the soft agar transformation and transwell metastasis assays, LMP1 enhances cell growth and migration through activation of phosphatidylinositol 3-kinase (PI3K)/Akt and NFκB signaling (Shair et al., 2009). LMP1 activates multiple distinct signaling pathways (EGFR, STAT3, and ERK) via PKCδ (Kung et al., 2011). This activation is seen not only in EBV infected cells but also in epithelial, endothelial, and fibroblast cells that receive purified exosomes from EBV infected cells. Downstream effects of this activation are expression of cytokines and chemokines leading to inflammation. Preparations of exosomes containing LMP1 were shown to inhibit the proliferation of peripheral blood mononuclear cells. In EBV-associated tumors such as NPC and HD, LMP1-containing exosomes may be taken up by infiltrating T-lymphocytes, where LMP1 could exert its anti-proliferative effect to allow the tumor cells to evade the immune system (Flanagan et al., 2003). LMP1 induces secretion of exosomes that contain fibroblast growth factor (FGF-2; Ceccarelli et al., 2007). Secretion of angiogenic factors such as FGF-2 may be responsible for the metastasis essential for tumor spread seen in EBV-associated cancers.

Galectin-9 is a protein that specifically interacts with membrane receptor Tim1 and induces apoptosis in T cells. Conditioned media from EBV infected xenografts which were either LMP1-positive or negative secreted exosomes containing galectin-9. These exosomes could inhibit T-cell proliferation assessed on peripheral blood mononuclear cells activated by CD3/CD28 cross-linking (Keryer-Bibens et al., 2006).

Deoxyuridine triphosphatase (dUTPase) is an enzyme that catalyzes hydrolysis of dUTP to deoxyuridylate and inorganic pyrophosphate. Mice injected with purified EBV dUTPase show inhibition of replication of mitogen-stimulated lymphocytes (Padgett et al., 2004). Ariza et al. (2013) observed at least a 700-fold enrichment of dUTPase activity in exosomal fractions when compared to concentrated culture supernatants from chemically induced Raji cells, and they found a 4.8-fold increase in dUTPase activity in exosomes derived from chemically induced Raji cells relative to non-induced cells. EBV-encoded dUTPase secreted in exosomes from chemically induced Raji cells induced the secretion of cytokines and chemokines through TLR2 in human primary recipient cells.

Elevated amounts of EGFR can be detected in NPC. EGFR expression and secretion from the cell in exosomes correlates with the levels of LMP1. In fact, LMP1 induces expression of EGFR (Meckes et al., 2010). Interferon-inducible protein 16 (IFI16) and cleaved caspase-1, IL-1β, IL-18, and IL-33 are all seen in the exosomes from Raji cells and EBV-transformed cell lines (Ansari et al., 2013). The expulsion of nuclear-resident protein IFI16 via exosomes is critical to EBV’s escape of recognition by the innate immune response since the protein functions as the non-sequence-specific direct recognizer of the EBV genome.

***Translational studies on EBV exosomes***

Since the EBV proteins LMP1 and BARF1 are detected in serum of EBV-infected patients, these proteins can serve as efficient diagnostic markers for NPC (Houali et al., 2007). LMP1 has greater potential for a biomarker than BARF1 has because LMP1 is found in exosomes that can easily be purified and provide a natural mechanism for concentration of the protein. In patients infected with EBV, EBV BART miRNAs are present in both B-cell and non-B-cell fractions, but EBV DNA is restricted to the circulating B-cell population, which suggests miRNA can be transfered via exosomes *in vivo* (Pegtel et al., 2010). Confirmation of this type of transfer came from studies by Gourzones et al. (2010), who demonstrated that the sera of NPC patients also contain BART miRNAs. Plasma samples from NPC patients and mice xenografted with NPC tumors also show presence of galectin-9-containing exosomes (Klibi et al., 2009). Incorporation into exosomes protects galectin-9 against proteolytic cleavage, retaining its Tim-3-binding capacity. In turn, these exosomes have been shown to induce massive apoptosis in EBV-specific CD4^+^ cells used as a model of target T cells.

***EBV miRNA in exosomes***

Pegtel et al. (2010) observed viral BHRF1 miRNAs in B95.8-immortalized EBV-immortalized cells that are delivered by exosomes to non-infected cells. The exosomes targeted the CXCL11/ITAC gene in uninfected neighboring cells. Gourzones et al. (2010) showed that EBV miR-BARTs present in exosomes can be detected in the serum of mice xenografted with human NPC cells. miR-BART15-3p was found highly enriched in exosomes from an EBV-positive gastric cancer cell line. miR-BART15-3p targets the apoptosis inhibitor BRUCE to induce apoptosis in recipient cells. This shows how, again, an exosomal component can provide a favorable microenvironment for the growth of EBV-associated tumors (Choi et al., 2013). However, not all EBV-encoded miRNA is transferred via exosomes since high concentrations of the viral microRNA BART17 have been detected outside of exosomes in plasma samples from NPC patients (Gourzones et al., 2013). Therefore exosomes have the potential to modulate immune functions of neighboring cells to alter the microenvironment and contribute to the pathophysiology of EBV-associated diseases because of the secretion of oncogenic, anti-apoptotic and pro-inflammatory molecules in exosomes from EBV infected cells.

#### Herpesvirus

The role of protein transfer from intracellular compartments to exosomes has also been studied in Herpes-simplex virus-1 (HSV-1) infected cells. Temme et al. (2010), have shown that the HSV-1 encoded glycoprotein B (gB) manipulates class II processing pathway by perturbing endosomal sorting and trafficking of HLA-DR (DR) molecules. Co-staining of gB, DR, and CD63 suggested that these markers are contained in enlarged vesicular structures of endosomal origin, and intervention of gB in this route appears to induce formation of enlarged vesicles. Interestingly, the human herpes virus 6 (HHV-6) induces formation of multivesicular bodies (MVBs; Mori et al., 2008). In ILVs contained in these MVBs, HHV-6–derived glycoproteins B and M were detected. HHV-6 uses the cellular exosomal pathway of the host cell for exiting the cell.

An attractive target for viruses is the ubiquitin system, which regulates intracellular sorting of proteins and their degradation. Ubiquitination was reported to be a signal for sorting of ILV in MVBs (Piper and Katzmann, 2007). Along these lines, Temme et al. (2010), found ubiquitination of gB in immunoprecipitates of the viral proteins from MJ-gB cells. Collectively, their data suggests that cellular ubiquitination of gB correlates with increased release of exosomal material further indicating that exosomes can transfer MHCII/peptide complexes to other cell types. The recipient cells then acquire the ability to present specific antigen. It is conceivable that a transfer of gB or of gB/DR complexes by exosomes from HSV-1–infected cells to other cells may also modulate immune responses to the viral antigens.

Enveloped virions are known to be released by the exosomal pathway. Mori et al. (2008) have shown that HHV-6-infected cells were larger than uninfected cells and contained many newly formed MVB-like compartments, including small vesicles that surround the Golgi apparatus. They also found that virus budding at TGN associated membranes, which expressed CD63, can incorporate adaptor protein (AP-1), TGN46, and CD63 into virions. Therefore, mature HHV-6 virions may be released together with internal vesicles through MVBs by the cellular exosomal pathway. Remarkably, the vacuoles that enwrapped the mature virions contained clathrin-coated membrane domains that frequently form a bud. This may be similar to human cytomegalovirus (HCMV) infection, where EM analysis shows viral particles within MVBs and occasionally budding into MVBs; therefore, MVBs might also be the final budding site of HCMV (Fraile-Ramos et al., 2002).

Finally, the role of MVBs may be that HHV-6 replicates more efficiently after virus spreading by direct cell-to-cell contact than after cell-free spread. It is conceivable that in T cells, the viral glycoproteins and cellular proteins expressed on exosomes may interact to form a “virological synapse” to promote the efficient spreading of virus from infected to uninfected cells.

***Kaposi’s sarcoma-associated herpesvirus***

There have been efforts to define not only contents of EBV and KSHV exosomes but also their function in recipient cells. For example, Meckes et al. (2013) showed that distinct viral-specific effects from KSHV exosomes would affect cellular metabolism, whereas EBV specific exosomes would activate cellular signaling mediated through integrins, actin, interferons (IFN), and NFκB. These findings suggested that both viruses affect exosome content to modulate both cell death and protein synthesis. KSHV recently has been shown to reprogram the host B-cell metabolism, promoting glycolysis, and the exosomes produced by the KSHV infected cells are highly enriched with proteins from the glycolytic pathway. Overexpression of pyruvate kinase, lactase dehydrogenase, or phosphoglucose isomerase increases glycolysis, and thus exosomal transfer of these enzymes could modulate metabolism in recipient cells. The KSHV exosomes also include proteins involved in remodeling epithelial adherens junctions, suggesting that these exosomes may modulate cell anchorage or movement. Therefore, it is likely that these effects contribute to viral persistence and pathogenesis.

Factors that could potentially act as a restriction factor in the lytic replicative cycles can also be found in exosomes from herpesvirus infections. For example, during primary KSHV infection of endothelial cells, acting as a nuclear pattern recognition receptor, gamma IFI16 colocalized with the KSHV genome in the nuclei and interacted with apoptosis-associated speck-like protein containing a CARD (ASC) and procaspase-1 to form a functional inflammasome (Singh et al., 2013). Release of IFI16 in the exosomes derived from BCBL-1 cells further suggested a higher-order complexity of viral regulation of host-mediated responses. A recent report describes how IFI16 acts as a restriction factor for HCMV replication (Gariano et al., 2012). The authors demonstrate that small interfering RNA-mediated silencing of IFI16 in human embryo lung fibroblasts (HELFs) result in enhanced replication of HSV-1, HSV-2, and HCMV (Gariano et al., 2012). Therefore IFI16 may be a novel restriction factor in the lytic replicative cycles of herpesviruses that is present in the nucleus to control viral DNA replication and also to be secreted within exosomes to control latency.

Finally, exosomes from KSHV infected cells serve as a means of intercellular communication with surrounding cells, and the contents of exosomes can be shared between cells through the mechanism of exosomal transfer. KSHV exosomes can deliver functional miRNAs to recipient cells to downregulate expression of target genes. In a recent study, Chugh et al. (2013) demonstrated that exosomes from patient PEL fluid, the BCBL1 PEL cell line, and a xenograft mouse model of KS confer an enhanced migration phenotype to hTERT-HUVEC cells *in vitro* as well as in a scratch assay. Their data implies that miRNAs specifically expressed within exosomes aid in disease progression and mediate paracrine effects, which are commonly seen in KSHV tumorigenesis (Chugh et al., 2013).

### RNA Viruses

#### HIV/HTLV Infection

Arenaccio et al. (2014) have shown that exosomes from HIV-1-infected primary CD4^+^ T lymphocyte are able to activate quiescent human primary CD4^+^ T lymphocytes, which can then replicate HIV-1. Nef, ADAM17, and TNF-α were part of the underlying mechanism. TNF-α release requires the activity of protease ADAM17, also called TNF-α-converting enzyme (TACE), which can be transferred/provided by exosomes. They also found that resting CD4^+^ T lymphocytes challenged by exosomes from HIV-1-infected cells release both TNF-α and IL-2. TNF-α release peaked at 6 h after exosome challenge, thereby remaining sustained within the 48 h of observation; whereas the highest number of IL-2-producing cells was present at the 48-h time point. These data suggested that the IL-2 release was a consequence of cellular activation induced by the autocrine/paracrine stimulus of TNF-α. The data implied a model where Nef expression was necessary to arm exosomes with active ADAM17 in order to render resting bystander lymphocytes permissive for HIV replication. This mechanism would be particularly relevant in primary infection and would explain the rapid spread of viral infection in organs including gut.

Similar to other pathogens, retroviruses hijack exosomal proteins as well as other components to increase their spread throughout the body. In addition to this benefit, the resulting changes in recipient cells can be profound leading to disease state and pathologies associated with an infection. To this end, HIV-1 infected macrophages have been shown to have increased number of exosomes and other vesicles secreted from the cell (Kadiu and Gendelman, 2011a,b; Kadiu et al., 2011, 2012). Importantly, these exosomes have been shown to contain various cytokines that induce migration and release of other inflammatory cytokines from recipient cells leading to enhanced HIV-1 infectivity. Moreover, some of the virions shed from infected macrophages have been shown to be associated with large aggregates of exosomes secreted from cells and resulting entrapment of virions has demonstrated enhanced infectivity toward target cells including T cells as compared to free purified virions. Additionally, the proteomic analysis of the exosomes from macrophages has identified proteins required for T cell activation, antigen presentation, and chemotaxis. Similar to these findings, we have also recently identified alteration of proteins and cytokines packaged into exosomes from HTLV-infected cells (Jaworski et al., 2014a). The release of these cytokines packaged into exosomes from HTLV-infected cells can lead to enhanced infectivity in recipient cells as well as neuroinflammation associated with HAM/TSP pathology. Therefore, collectively these data demonstrate multiple potential mechanisms where virally-infected cells control exosome release to assist in viral spread and induction of pathologies. These vesicles can in turn activate naïve cells leading to better uptake of virus and cell to cell transmission of viral particles through viral synapses.

In addition to changes in the composition of host proteins both HIV and HTLV readily incorporate their own nucleic acids and viral proteins into the exosomes. For instance, recent development has shown that Tax of HTLV-1 is incorporated into the exosomes, which are secreted from both cell lines and primary cells infected with HTLV-1 (Jaworski et al., 2014a). The release of Tax in these exosomes represents one mechanism where HTLV-1 virus can induce deregulation of T cells as well as increase in neuroinflammation observed in HAM/TSP patients. Additionally, while the overall mechanism of trafficking of viral proteins in HTLV-1 is not well defined, on the other hand HIV-1 exosomes incorporate Nef, which represents a well-defined biochemical pathway of trafficking of proteins into microvesicles.

Beyond protein changes observed in the exosomes, there are also small RNA molecules that are packaged and incorporated into exosomes from infected cells. For instance, we have recently found that a 52-base stem-and-loop structure called TAR (viral transacting response element) transcribed from integrated provirus is incorporated within exosomes from HIV-1 infected cells. These TAR element is found not only in cell culture, but also sera of patients on antiretroviral drugs as well as long-term non-progressors (LNTPs; Narayanan et al., 2013). The concentration of the TAR molecule in these vesicles are high enough that simple PCR methods can be used to detect RNA from patient sera (103–106 copies per ml). Furthermore, the TAR RNA was found to be associated with components of the miRNA machinery which are involved in generation of specific 5′ and 3′ miRNAs. Interestingly, the level of components of the miRNA machinery incorporation into exosomes varies dramatically between HIV-1 and HTLV infected exosomes. For instance, we have recently found that in contrast to HIV-1 infected cells exosomes from cell lines infected with HTLV-1 contain Tax protein but minimal to no viral miRNA (Jaworski et al., 2014a). There is also an increased level of Ago2 bound to cellular miRNAs suggesting that HTLV-1 infected exosomes can rapidly control mRNA and their subsequent inhibition in recipient cells.

We have also recently observed presence of full-length viral RNA in the exosomes from HIV-infected cells when using nanoparticle capture assays (described above). These exosomes have minimal capacity for infection in recipient cells leading us to believe that genomic RNAs that may be packages in the exosomes (CD63 positive exosomes) are not infectious primarily due to lack of all the components necessary for the reverse transcription step. Collectively exosomes secreted from HIV-1 and HTLV-1 infected cells allow activation of recipient cells and subsequent exposure to the virus resulting in enhanced pathology.

#### Hepatitis C Virus

The hepatitis C virus (HCV) is a small enveloped virus with positive-sense single-stranded RNA genome which belongs to the family *Flaviviridae* (Cuthbert, 1990). This virus infects hepatocytes where the viral RNA replicates in the cytoplasm via the viral RNA-dependent RNA polymerase. This enzyme produces a negative-strand RNA that serves as a template for new positive-strand viral genomes that can then be translated or packaged into the new viral particles. The virus replicates on the membranes of endoplasmic reticulum (Dubuisson et al., 2002). Nascent virus particles use the cellular secretory pathway for budding and release. The progeny HCV particles consist of a nucleocapsid containing the viral genome, enveloped by an endoplasmic reticulum-derived lipid bilayer with viral envelope glycoproteins (Catanese et al., 2013). However, the level of virion production in HCV infected cells is very low; virions are unstable and structurally irregular (Catanese et al., 2013). Recent studies suggest that HCV virion assembly and release in hepatocytes can be associated with the exosome secretory pathway (Tamai et al., 2012). Thus, the assembly pathway engaged by this virus leads to incorporation of whole virions or only nucleocapsids, envelope proteins and replication-competent viral RNA into the exosomes (Masciopinto et al., 2004; Ramakrishnaiah et al., 2013; Bukong et al., 2014; Liu et al., 2014; Longatti et al., 2014). Recent studies of Ramakrishnaiah et al. (2013) showed that apart from classical transmission by free viral particles, HCV can be transmitted by exosomes to naïve human hepatoma Huh7.5.1 cells and can establish a productive infection with efficiency comparable to that established by free infectious particles. Because of this transmission mechanism, the virus can evade humoral immune responses (Bruggmann et al., 2008; Fafi-Kremer et al., 2010). Analysis of the exosomes from HCV-infected hepatoma cells revealed presence of full-length viral RNA along with host-cell RNA molecules, including miR122 (Pan et al., 2012; Bukong et al., 2014). Viral envelope E1 and E2 proteins were shown to be unnecessary for the transmission of exosomes, albeit incorporation of these glycoproteins into the exosomes led to enhanced transmission (Ramakrishnaiah et al., 2013).

Later studies of Bukong et al. (2014) demonstrated the capacity of the exosomes isolated from sera of chronic HCV infected patients to mediate viral receptor-independent transmission of HCV to naïve hepatocytes. The exosomes contained negative-strand HCV RNA, indicating incorporation of replication competent viral genome. The exosomes containing the HCV genome also harbored miR-122, as well as Ago2 and HSP90 proteins. Earlier studies demonstrated that these components may enhance HCV replication (Jopling et al., 2005; Henke et al., 2008; Wilson et al., 2011). Consistent with recent reports, Ago2 and miR-122 can enhance HCV replication when bound to the 5′-UTR of HCV dsRNA (Wilson et al., 2011); miR-122 was demonstrated to be present in exosomes isolated from both HCV infected Huh7.5 cells and HCV-infected individuals. Bukong et al. (2014) found that Ago2 is associated with miR-122, positive sense HCV RNA and, in some cases, negative sense HCV RNA as well. Using co-immunoprecipitation, they confirmed that HSP90 and Ago2 form complexes within the HCV containing exosomes, likely to provide further stabilization of the HCV RNA-replication complex (Bukong et al., 2014). This shows that miR-122 in the exosomes can provide advantages for HCV transmission (Bukong et al., 2014). Taken together, existing data indicate that the exosomes from HCV infected cell line, as well as serum exosomes from some HCV infected treatment-naïve patients contain positive sense viral RNA and are able to transmit active HCV infection.

### Bacterial Pathogens

Pathogen-associated molecular patterns have been identified on exosomes in multiple studies (Fleming et al., 2014). These molecules are frequently lipid- and/or carbohydrate-containing molecules. For example, mycobacterial components such as lipoarabinomannan (LAM) as well as mycobacterial proteins were found in exosomes (Beatty et al., 2000) and these exosomes can bind to normal, uninfected cells (Beatty et al., 2000, 2001; Bhatnagar et al., 2007), possibly by binding to PAMP-receptors such as toll like receptors (O’Neill and Quah, 2008). Exosomes containing certain PAMPs are also capable of stimulating a proinflammatory response in uninfected cells (Bhatnagar and Schorey, 2007; Colino and Snapper, 2007; Singh et al., 2012). For example, exosomes from *Salmonella typhimurium*-infected macrophages, which were found to contain *Salmonella* lipopolysaccharide, stimulated proinflammatory responses both in cell-based assays and in mice (Bhatnagar et al., 2007). *Mycobacterium tuberculosis*-infected macrophages also had this effect (Bhatnagar and Schorey, 2007) while simultaneously triggering the downregulation of a number of IFN-γ inducible genes in naïve cells including inhibition of inducible expression of the MHC-II and CD64 immune receptors (Singh et al., 2011). Exosomes from cells infected with the common cell culture contaminant *Mycoplasma* genus of intracellular parasitic bacteria have been shown to induce activation of B cells (Quah and O’Neill, 2007), which in turn actively inhibit T cell activation (Yang et al., 2012a). The activation of immune cells by exosomes released from *M. tuberculosis* infected cells has also been demonstrated *in vivo* in the absence of infection (Giri et al., 2010). Recently, the laboratory of one of the authors (RMH) has found that exosomes released from cells infected with *Yersinia pestis* induce cytokine release in naïve cells, and that the treated cells also demonstrate a phenotype that is similar to infection with *Y. pestis*. Studies are currently under way to evaluate the contributions of these exosomes to the process of pathogenesis.

In the case of *B. anthracis* infection, exosomes from infected cells have been shown to contain the lethal toxin (LT) virulence factor, allowing for the delivery of LT to cells at sites distal to infection (Abrami et al., 2013). LT consists of two components, the pore forming protective antigen (PA) and the metalloprotease lethal factor (LF). After LF is translocated into the cytoplasm by PA, it is then able to be translocated into ILVs by PA. This not only provides a location for LF to avoid proteolytic degradation once inside cells, but also allows for the release of host-derived exosomes containing LF, which likely contributes to the persistence and spread of the toxins inside the host (Abrami et al., 2013). Similarly, exosomes released by cells after infection with *Staphylococcus aureus* have been shown to contain the bacterial pore forming α-toxin, also allowing for delivery of bacterial virulence factors to cells removed from the immediate area of infection (Husmann et al., 2009). Cells targeted by the β-barrel forming α-toxin are able to endocytose areas of their cell membrane containing the toxin to avoid cell lysis. These endocytosed vesicles containing the α-toxin are trafficked to the late endosome where they avoid degradation and are instead incorporated in exosomes secreted from the host cell. These secreted exosomes contain both multimeric and monomeric toxin, both of which could potentially be active when taken up by naïve cells. Taken together these examples suggest a mechanism by which exosomes containing or presenting PAMPs may contribute to pathogenesis in the host, potentially at a distant site from the primary infection.

### Protozoan and Helminth-Derived Exosomes

Protozoan parasites provide multiple examples of pathogen-derived molecules in exosomes (Aline et al., 2004; Beauvillain et al., 2009; Schnitzer et al., 2010; Hu et al., 2013). A protease (GP63 surface protease) of *Leishmania mexicana* has been identified on exosomes (Hassani and Olivier, 2013), possibly suggesting that an enzymatic activity (protease) could be transmitted to distant sites by this mechanism. *T. gondii* infection leads to the inclusion of *T. gondii* molecules in exosomes (Aline et al., 2004; Bhatnagar et al., 2007; Beauvillain et al., 2009). Another protozoan, *Cryptosporidium parvum*, has recently been found to strongly induce increased production of exosomes from both biliary and intestinal epithelium as a result of infection (Hu et al., 2013), which could then increase the host-response to any PAMP-molecules that might be included in those exosomes.

Thus, PAMPs have been identified on exosomes from multiple viral, bacterial and protozoan pathogens, suggesting that this might be a more general phenomenon of host-response to infection (Fleming et al., 2014). Advances in our understanding of the PAMPs in exosomes could potentially lead to the development of exosome-based diagnostic tests which could be readily performed on blood, saliva or urine samples (Wang and Sun, 2014; Zheng et al., 2014; Huebner et al., 2015), which have been shown to contain exosomes.

Helminths are parasitic worms including nematodes (roundworms), tapeworms and flatworms that can cause serious and chronic infectious diseases such as schistosomiasis and hookworm. In particular, soil-transmitted helminthiasis, schistosomiasis, and lymphatic filariasis are considered neglected tropical diseases (Prichard et al., 2012). It has been estimated that up to one-third of the three-billion impoverished humans (those who live on less than $2 US per day) may be infected with one or more species of helminths (Hotez et al., 2008), and these helminth infections may also be co-endemic with malaria and HIV/AIDS in these populations. According to the WHO online factsheet N°366, as many as 1.5 billion people, or 24% of the globe’s population may be infected with soil-transmitted helminth infections.

Exosomes of endocytic origin and typical exosome size (30–100 nm) have been identified that contain helminth parasite proteins. As an example, Marcilla et al. (2012) have demonstrated the presence of exosomes produced from two trematodes (*Echinostoma caproni* and *Fasciola hepatica*) have shown that these EVs are “actively released” and demonstrated that they can be taken up by host cells. The identification of exosomes in these organisms also explained a phenomenon that had been previously observed in trematodes, called “secretion of atypical proteins” (Marcilla et al., 2012). Bernal et al. (2014) recently identified 84 helminth proteins in *Dicrocoelium dendriticum*-derived exosomes by proteomic analysis, as well as identifying helminth miRNA within the exosomes, which may be the first report of miRNA detected in helminth-derived exosomes.

A nematode parasite of the murine gastrointestinal tract, *Heligmosomoides polygyrus*, was shown to also produce exosomes that contain nematode proteins homologous to mammalian exosome proteins, as well as miRNA, and a nematode homolog of Argonaute protein (Buck et al., 2014). Furthermore, in this study, helminth-derived exosomes were found to suppress murine Type 2 innate immune response, mediated by the nematode miRNA in the exosomes (Buck et al., 2014). In a related study, *H. polygyrus*-derived exosomes were shown to induce a murine host response when administered (Marcilla et al., 2014; Montaner et al., 2014), suggesting that helminth-derived exosomes are able to significantly modulate the host immune response to the parasitic infection.

Collectively, these studies suggest an emerging and important role for helminth-derived exosomes in host-parasite communication. In addition, proteins and molecules identified on and in helminth-derived exosomes could provide the basis for new diagnostic markers for helminth infections. Thus, there is an increasingly better defined role for EVs including exosomes in the helminth parasite’s interaction with their hosts. Given that there are more than 300 species of human-parasitizing helminths, there will likely be many more examples of EVs being produced from this group, and demonstrations of their complex interactions with their eukaryotic hosts (Marcilla et al., 2014).

## Conclusion

Exosomes, as well as other types of vesicles, are released from the cell membrane from many cell types in the body after forming in late endosomes. These vesicles contain proteins, RNA, and lipids from the host cell. Several databases are available with information on the thousands of proteins and RNA that have been found in exosomes including specific markers that can be used to purify them from supernatant and other bodily fluids including Alix and CD63.

Among DNA viruses, virally-infected cells can secrete viral proteins and RNA as well as cellular proteins and miRNA in exosomes. Recently, researchers have found that EBV miRNA and functional viral proteins such as LMP1 in exosomes from infected cells cause cell growth and migration by activating the PI3K/Akt pathway. Galectin-9 and dUTPase are 2 other viral proteins that can spread through exosomes and cause apoptosis and inflammation, respectively. HHV-6 infected cells release virions enveloped in an exosome, which leads many to believe that the virus spreads through cell-to-cell contact. KSHV exosomes cause changes in the recipient cell metabolism and aid in latency. The miRNAs from KSHV exosomes have shown to aid in tumorigenesis.

RNA viruses such as HIV-1 infected macrophages release an increased number of exosomes as compared to non-infected cells, as the virus utilizes the endosomal pathway to spread infection. Cytokines are incorporated into exosomes from HIV-1 or HTLV-infected cells that can cause cell migration in recipient cells, and therefore faster spread of infection. The cytokine expression is further controlled by either cellular or viral non-coding RNAs such as HIV-1 TAR. HCV may also utilize the exosomal pathway to incorporate the nucleocapsid or whole virion into exosomes.

In addition to virally-infected host cells, bacteria, protozoa, and fungi can all release small vesicles into the extracellular environment. Bacteria release small vesicles that contain PAMPs, which signal to uninfected cells to release proinflammatory cytokines. Protozoan parasites also release exosomes that cause the release of subsequent cellular exosomes controlling signal transduction and cell death. Exosomes released from helminthes and contain helminth proteins can be taken up by host cells.

Exosomes can be used to protect against further infection, as shown in animal models by activating CD4^+^ T cells, and can be modified into effective vaccines. Finally, exosomes can be modified into biotherapeutics that can easily pass through the blood–brain barrier. This concept has been shown with curcumin. Other ongoing studies focus on targeting the exosomes for specific tissue types by modifying surface proteins. This method has been proven on DC-derived exosomes targeting the brain. Collectively, the field of exosome research holds much promise in not only understanding new and novel conceptual and fundamental pathways in cellular signal transduction, but also how infectious diseases utilize this pathway to contribute to pathogenesis.

### Conflict of Interest Statement

The authors declare that the research was conducted in the absence of any commercial or financial relationships that could be construed as a potential conflict of interest.
